# Biomimetic Synthesis of Selenium Nanospheres by Bacterial Strain JS-11 and Its Role as a Biosensor for Nanotoxicity Assessment: A Novel Se-Bioassay

**DOI:** 10.1371/journal.pone.0057404

**Published:** 2013-03-04

**Authors:** Sourabh Dwivedi, Abdulaziz A. AlKhedhairy, Maqusood Ahamed, Javed Musarrat

**Affiliations:** 1 Department of Zoology, College of Science, King Saud University, Riyadh, Saudi Arabia; 2 King Abdullah Institute for Nanotechnology, King Saud University, Riyadh, Saudi Arabia; 3 Department of Agricultural Microbiology, Faculty of Agricultural Sciences, Aligarh Muslim University, Aligarh, India; Aligarh Muslim University, India

## Abstract

Selenium nanoparticles (Se-NPs) were synthesized by green technology using the bacterial isolate *Pseudomonas aeruginosa* strain JS-11. The bacteria exhibited significant tolerance to selenite (SeO_3_^2−^) up to 100 mM concentration with an EC_50_ value of 140 mM. The spent medium (culture supernatant) contains the potential of reducing soluble and colorless SeO_3_^2−^ to insoluble red elemental selenium (Se^0^) at 37°C. Characterization of red Se° product by use of UV-Vis spectroscopy, X-ray diffraction (XRD), atomic force microscopy (AFM) and transmission electron microscopy (TEM) with energy dispersive X-ray spectrum (EDX) analysis revealed the presence of stable, predominantly monodispersed and spherical selenium nanoparticles (Se-NPs) of an average size of 21 nm. Most likely, the metabolite phenazine-1-carboxylic acid (PCA) released by strain JS-11 in culture supernatant along with the known redox agents like NADH and NADH dependent reductases are responsible for biomimetic reduction of SeO_3_^2−^ to Se° nanospheres. Based on the bioreduction of a colorless solution of SeO_3_^2−^ to elemental red Se^0^, a high throughput colorimetric bioassay (Se-Assay) was developed for parallel detection and quantification of nanoparticles (NPs) cytotoxicity in a 96 well format. Thus, it has been concluded that the reducing power of the culture supernatant of strain JS-11 could be effectively exploited for developing a simple and environmental friendly method of Se-NPs synthesis. The results elucidated that the red colored Se° nanospheres may serve as a biosensor for nanotoxicity assessment, contemplating the inhibition of SeO_3_^2−^ bioreduction process in NPs treated bacterial cell culture supernatant, as a toxicity end point.

## Introduction

Selenium (Se^0^) is a trace element commonly found in materials of the earth’s crust, and belongs to group 16 (chalcogens) of the periodic table. Se° is well known for its photoelectric, semiconductor, free-radical scavenging, anti-oxidative and anti-cancer properties [1]. It occurs in different forms as red amorphous selenium (Se^0^), highly water soluble selenate (SeO_4_^2**−**^) and selenite (SeO_3_^2**−**^), and as gaseous selenide (Se^2**−**^). Amongst its various forms, the SeO_3_^2**−**^ is highly toxic, which adversely affect the cellular respiration and antioxidant system causes protein inactivation and DNA repair inhibition [2], [3], [4]. Therefore, detoxification of SeO_3_^2**−**^ has attracted a great deal of attention, particularly the reduction of this oxyanion by the microorganisms. The SeO_3_^2**−**^ reducing bacteria are ubiquitous in diverse terrestrial and aquatic environments [5]. The ability to reduce the toxic SeO_4_^2**−**^ and SeO_3_^2**−**^ species into non-toxic elemental form Se° has been demonstrated under aerobic and anaerobic conditions [5], [6], [7], [8]. However, the reduction of SeO_3_^2**−**^ to Se^0^, which is a common feature of many diverse microorganisms, is still not well understood. Earlier studies have suggested that SeO_3_^2**−**^ reduction may involves the periplasmic nitrite reductase [8], [9] in *Thauera selenatis* [10] and *Rhizobium selenitireducens* strain B1 [11], [12], [13], nitrate reductase in *E. coli* [14], hydrogenase I in *Clostridium pasteurianum* [15] and arsenate reductase in *Bacillus selenitireducens* [16] or some of the non-enzymatic reactions [17]. Lortie et al. [6] have reported aerobic reduction of SeO_4_^2**−**^ and SeO_3_^2**−**^ to Se° by a *Pseudomonas stutzeri* isolate. Studies based on X-ray absorption spectroscopy also revealed that the soil bacterium *Ralstonia metallidurans* CH34, resistant to SeO_3_^2**−**^ is capable of its detoxification, and localize the red Se° granules mainly in the cytoplasm [18]. Sarret et al. [19] investigated the kinetics of selenite and selenate accumulation and Se speciation to identify the chemical intermediates putatively appearing during reduction using X-ray absorption near-edge structure (XANES) spectroscopy. Furthermore, the NADPH/NADH dependent selenate reductase enzymes have been reported to catalyze the reduction of selenium oxyions [20], [21]. Most of the studies on the biogenesis of selenium nanoparticles (Se-NPs) are based on anaerobic systems. However, there are also few reports in literature on the aerobic formation of Se-NPs by microorganisms such as *Pseudomonas aeruginosa*, *Bacillus sp.* and *Enterobacter cloacae* [22], [23], [24].

With the overwhelming growth in the field of nanotechnology and a rapid stride in the synthesis and commercialization of nanomaterials, the occupational and inadvertent exposure to human population is imminent, which may pose serious hazards to human health and ecosystem. Therefore, improved characterization and reliable toxicity screening tools are required for exposure risk assessments. The commonly used cytotoxicity screening assays are mostly based on fluorescence or absorbance measurements following toxicant exposure and incubation with a colorimetric indicator dyes but has many limitations with nanotoxicity assessment [25], [26], [27], [28].Thus, Wang et al. [29] developed a novel bioluminescence inhibition assay exploiting *Photobacterium phosphoreum* to evaluate the toxicity of quantum dots. Also, a black and white method (Te-assay) for pre-screening of environmental samples based on reduction of tellurite (TeO_3_^2**−**^) to elemental tellurium has been reported [30]. Along the similar principle, we have attempted to exploit the selenite tolerant *Pseudomonas aeruginosa* strain JS-11 isolated from wheat rhizosphere for biosynthesis of Se-NPs and utilized its capacity of reducing of SeO_3_^2**−**^ to Se^0^, as a metabolic marker for visual assessment of the relative toxicity of several NPs in a single experiment in a 96-well format. Thus, the objectives of the study were to investigate the (i) metabolic potential of a SeO_3_^2**−**^ tolerant *P. aeruginosa* strain JS-11 for green synthesis of elemental Se° nanospheres, (ii) characterization of Se-NPs by use of UV–Vis spectrophotometry, X-ray diffraction (XRD), dynamic light scattering, transmission electron microscopy (TEM), energy dispersive X-ray (EDX) analysis, Fourier transform infra red spectroscopy (FTIR) and atomic force microscopy, and (iii) development of a simple, colorimetric assay for toxicity assessment of NPs and other environmental pollutants.

## Materials and Methods

### Bacterial Growth and Resistance to SeO_3_^2**−**^ Stress

The soil bacteria *P. aeruginosa* strain JS-11, initially isolated in our laboratory from wheat rhizosphere of herbicide contaminated soil by the enrichment culture technique [31], and maintained as glycerol cultures at −80°C, were used in this study. The strain JS-11 has already been well characterized based on its metabolic profile using BIOLOG GN plates (Biolog Inc., Hayward, CA, USA) and phylogenetic analysis based on 16SrDNA sequence homology [31]. In order to assess the tolerance of strain JS-11 for SeO_3_^2**−**^ and its reduction to Se^0^, the frozen culture was thawed and grown in Luria-Bertani (LB) broth. Cells from exponentially grown culture were streaked on to Luria agar (LA) plates supplemented with 12.5 mM sodium selenite (Na_2_SeO_3_^2**−**^). The red color colonies developed on the plates after 18 hours (h) of incubation at 37°C were transferred to fresh LB medium containing 25 mM Na_2_SeO_3_^2**−**^, and further sub-cultured for determining the SeO_3_^2**−**^ tolerance limit. The effect of SeO_3_^2**−**^ on bacterial growth was determined by culturing the cells (∼2×10^4^ CFUm1**^−^** ^l^) in 250 ml Erlenmeyer flasks containing 100 ml of LB supplemented with increasing concentrations (12.5, 25, 50, 100 and 200 mM) of Na_2_SeO_3_^2**−**^. The flasks were incubated at 37°C under constant shaking at 200 rpm for 24 h. The growth in each flask was determined by measuring the optical density (O.D.) at 600 nm. For growth kinetics, the bacterial growth was determined in the LB and M9 mineral salt medium (Na_2_HPO_4_.7H_2_O, 42 mM; KH_2_PO_4_, 24 mM; NaCl, 9 mM; NH_4_Cl, 19 mM; MgSO_4_, 1 mM; CaCl_2_, 0.1 mM and glucose 2%) supplemented with 12.5 mM Na_2_SeO_3_^2**−**^, as a function of time of incubation and the O.D. was measured periodically at 600 nm.

### Determination of Phenazine Production by Strain JS-11 in Culture Supernatant

The phenazine-1-carboxylic acid (PCA) production in bacterial cell culture was determined following the method of Mavrodi et al. [32]. In brief, the cell culture of strain JS-11 was grown at 37°C in a 250 ml conical flask containing modified LB medium (LB +1 mM tryptophan) that favors the PCA production. Tryptophan facilitates the synthesis of PCA via anthranilate synthase II pathway using anthranilate as a substrate [33], [34]. The culture was grown for 72 h and the cells were centrifuged at 5000 rpm for 10 minutes (min.). The cell-free culture supernatant (100 ml) was transferred to another flask and acidified with concentrated hydrochloric acid to achieve a pH of 2.0. The acidified supernatant was extracted with equal volume of benzene. The organic phase was pooled and dried by evaporation. The dried pale yellow residue was dissolved in 1 ml of 0.1 M NaOH, and the absorbance was read at 367 nm against the benzene extract of acidified LB alone, used as a blank. Further, the HPLC analysis of PCA extract was performed by use of Waters HPLC System coupled with 2487 dual λ UV/visible detector using C-18 Novapak (4 µm) column (Waters Corp., Milford, MA, USA) with mobile phase of acetonitrile: water (70∶30) at 254 nm. Fourier transform infra-red (FTIR) spectroscopic analysis was performed for examining the functional groups of PCA. The PCA was mixed with spectroscopic grade potassium bromide (KBr) in the ratio of 1∶100 and spectrum recorded in the range of 400–4000 wavenumber (cm^−1^) on FTIR spectrometer, Spectrum 100 (Perkin Elmer, USA) in the diffuse reflectance mode at a resolution of 4 cm^−1^ in KBr pellets.

### Determination of Reducing Activity in Culture Supernatant by KMnO_4_ Titrimetric Assay

Freshly grown cell of bacterial strain JS-11 were sub-cultured in LB broth containing the suspensions of silver nanoparticles (Ag-NPs, 27 nm), cadmium sulfide nanoparticles (CdS-NPs, 4 nm), titanium dioxide nanoparticles (TiO_2_-NPs, 30.6 nm) and zinc ferrite nanoparticles (ZnFe_2_O_4_-NPs, 19 nm) at two different concentrations of 50 and 100 µgml**^−^** ^1^. The cultures of untreated and treated bacterial cells were grown for 24 h at 37°C. Cultures were centrifuged at 5000 rpm for 10 min and the supernatants of untreated and NPs treated bacteria were assessed for the innate reducing activity based on potassium permanganate (KMnO_4_) back-titration, following the method of Fesharaki et al. [35]. Briefly, the supernatant (6 ml) was diluted by ultrapure water (20 ml) and acidified with 2 ml of 1.5 N phosphoric acid. The acidified supernatant was then oxidized with an excess of KMnO_4_ (0.1 N) for 30 min at 60°C. The unreacted permanganate was titrated with a 0.04 N oxalic acid solution. The end-point was determined with the disappearance of violet color of the reactant solution, and the reducing activity of the supernatants was calculated considering the volume and normality of permanganate solution. A parallel set of experiment was performed with the un-inoculated culture medium for data normalization, and results reported as mg of KMnO_4_ per ml of the supernatant.

### Fluorescence Measurements

Fluorescence spectra of the supernatants obtained from the untreated bacterial culture and those treated with 100 µgml**^−^** ^1^ of Ag-NPs, CdS-NPs, TiO_2_-NPs and ZnFe_2_O_4_-NPs were measured in a 1 cm path length cell by use of Shimadzu spectrofluorophotometer, model RF5301PC (Shimadzu Scientific Instruments, Japan) equipped with RF 530XPC instrument control software, at ambient temperature. The excitation and emission slits were set at 5 nm each. The emission spectra were recorded in wavelength range of 290–380 nm and the excitation wavelength was set at 280 nm. The Na_2_SeO_3_^2**−**^ solution was non-fluorescent at this wavelength range. The NADH fluorescence in the culture supernatant was also measured at 340 nm and 440 nm, as excitation and emission wavelengths, respectively.

### Biosynthesis of Elemental Se-NPs

The cells of bacterial strain JS-11 were grown in LB broth in a 500 ml flask at 37°C with agitation at 200 rpm. After 24 h of incubation, the cell culture was centrifuged at 5000 rpm for 10 min. The supernatant was transferred to 250 ml flask to which Na_2_SeO_3_^2**−**^ was added at a final concentration of 2 mM, and again incubated at 37°C, under constant agitation at 200 rpm for 72 h. The red colored (Se^0^) product obtained in the supernatant was recovered and analyzed for the presence of Se-NPs.

### Characterization of Se-NPs

#### UV–Visible spectral analysis

Color changes in the culture supernatant of strain JS-11 were monitored both by visual inspection and absorbance measurements using double beam UV–Vis spectrophotometer, (Labomed, U.S.A) as described earlier [36]. The spectra of the surface plasmon resonance of Se-NPs in the supernatants were recorded periodically at 2, 24, 48 and 72 h in wavelength range of 200 and 800 nm.

#### X ray diffraction analysis

The red colored bacterial culture supernatant containing Se° in the form of Se-NPs was freeze-dried on Heto Lyophilizer (Heto-Holten, Denmark) and stored in lyophilized powdered form until used for further characterization. The finely powdered sample was analyzed by X’pert PRO Panalytical diffractometer using CuK_α_ radiation (λ = 1.54056 Å) in the range of 20°≤2θ≤80° at 40 keV. In order to calculate the particle size (D) of the sample, the Scherrer’s relationship (D = 0.9 λ/βcosθ) has been used [37], where λ is the wavelength of X-ray, β is the broadening of the diffraction line measured half of its maximum intensity in radians and *θ* is the Bragg’s diffraction angle. The particle size of the sample was estimated from the line width of the (101) XRD peak.

### Transmission Electron Microscopic (TEM) and Energy Dispersive X-ray (EDX) Analysis

Samples for TEM analysis were prepared by drop-coating Se-NPs solution onto carbon-coated copper TEM grids. The films on the TEM grids were allowed to stand for 2 min. The extra solution was removed using a blotting paper and the grid dried prior to measurement. Transmission electron micrographs were obtained on JEM-2100F (JEOL Inc., Japan) instrument with an accelerating voltage of 80 kV. To ascertain the reduction of SeO_3_^2**−**^ to elemental selenium (Se^0^), the samples were processed by a method similar to that used for TEM studies. The selected areas within TEM sections were subjected to elemental composition analysis using an EDX (JEOL Inc., Japan).

### Dynamic Light Scattering and Zeta (ζ) Potential

Se-NPs powder was suspended in deionized ultrapure water to obtain a concentration of 50 µgml**^−^** ^1^, and sonicated at 40 W for 15 min. Hydrodynamic particle size and Zeta (ζ) potential of Se-NPs in an aqueous suspension were determined by measuring the dynamic light scattering by use of a ZetaSizer-HT (Malvern, UK).

### Atomic Force Microscopic (AFM) Analysis

Bacterial Se-NPs were examined using Innova AFM (Veeco Instruments, Plainview, NY, USA) in a non-contact tapping mode, following the method described by Musarrat et al. [36]. The topographical images were obtained in tapping mode at a resonance frequency of 218 kHz. Tapping mode imaging was implemented in ambient air by oscillating the cantilever assembly at or near the cantilever’s resonant frequency using a piezoelectric crystal. Characterization was done by observing the patterns on the surface topography and data analysis through WSXM software.

### Selenium Reduction Bioassay (Se-Assay) for Toxicity Assessment

The bacterial strain JS-11 was grown for 24 h at 37°C. Freshly grown culture (50 ml) was centrifuged at 4500 rpm for 10 min. The culture supernatant (1 ml) was then aliquoted into 1.5 ml eppendorf tubes for treatment with toxicants. To each tube, increasing concentrations (6.25, 12.5, 25, 50, 75, 100 µgml**^−^** ^1^) of various analyte NPs viz. Ag-NPs, CdS-NPs, TiO_2_-NPs and ZnFe_2_O_4_-NPs were added. A well known genotoxicant ethyl methane sulphonate (EMS) in concentration range of 0.125 - 2.0 mM, was used as a positive control. The tubes were incubated at 37°C for 24 h. Treated supernatants were again centrifuged for 10000 rpm for 10 min. Supernatants were collected separately in fresh tubes and 100 µl of each supernatant was then carefully transferred to all the wells (columns 1–12, rows A–F) in a 96-well microtitre plate. Subsequently, 100 µl of Na_2_SeO_3_^2**−**^ solution (final concentration 20 mM) was added to each well, except the Lane 1 (untreated control). The mixture was then incubated at 37°C for 48 h. The plate was read at 520 nm on multi-well microplate reader (Thermo Scientific, USA). For quantitative assessment, the intensity of red color in wells of all lanes (columns 5–12) was compared with the intensity of red color in the control lanes 2 and 3 (wells F2 and F3) containing cell-free culture supernatant+SeO_3_^2**−**^, by considering their mean O.D. value as 100%. The decrease in redness by 50% upon treatment with the increasing concentrations of analytes (NPs/EMS), provided the IC_50_ of the microbial activity, which refers to 50% loss of metabolic activity compared to the control/no-analyte cells.

## Results and Discussion

### Bacterial Tolerance to SeO_3_^2**−**^ in Culture Medium

**Figure 1:**
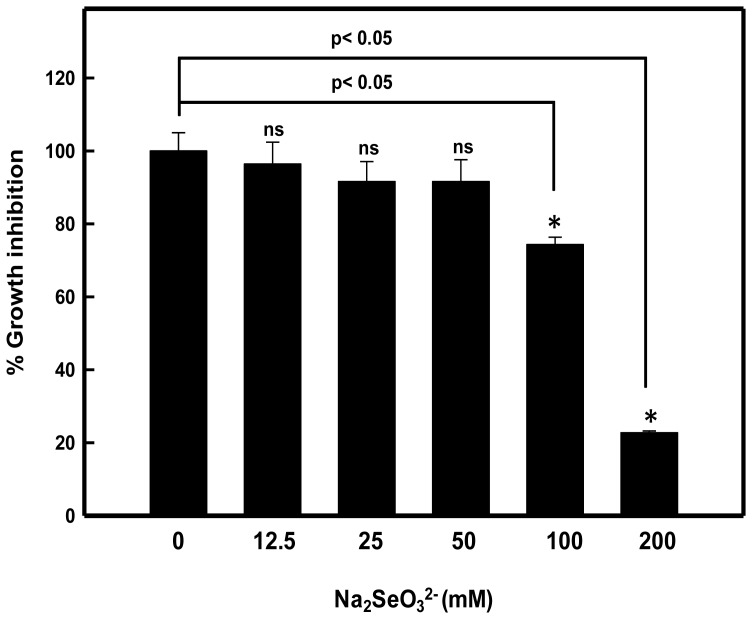
Selenite tolerance by *Pseudomonas aeruginosa* strain JS-11.

The results in Fig. 1 show the SeO_3_^2**−**^ tolerance of the *P. aeruginosa* strain JS-11 at increasing concentrations of Na_2_SeO_3_^2**−**^. The bacteria exhibited substantial growth in LB medium supplemented with Na_2_SeO_3_^2**−**^ in concentration range of 12.5 to 100 mM. Significant intensity of red color developed in culture medium after 24 h of growth due to reduction of SeO_3_^2**−**^ to elemental Se^0^, which suggested adequate metabolic activity in selenium oxyanions treated cells, as an indication of cell viability. The presence of SeO_3_^2**−**^ up to 50 mM resulted in 8.5% growth inhibition, whereas 25.7% (p<0.05) and 77.3% (p<0.05) growth inhibition was observed at 100 and 200 mM SeO_3_^2**−**^ concentrations, respectively, as compared to untreated control. Based on the extent of growth inhibition, the effective concentration (EC_50_) of SeO_3_^2**−**^ was determined to be 140 mM. Hunter and Manter [21] have also reported 57% and 66% reduced growth of *Pseudomonas* sp. strain CA5 at SeO_3_^2**−**^ concentrations of 100 and 150 mM, respectively. Therefore, our results explicitly suggested the *P. aeruginosa* strain JS-11, as SeO_3_^2**−**^ tolerant bacteria. Several earlier studies have suggested biogeochemical cycling of Se° through SeO_4_^2−/^SeO_3_^2**−**^ reduction with the mixed cultures or sediment samples [38], [39]. Furthermore, a decreased SeO_3_^2**−**^ reduction by *Pseudomonas strutzeri* isolate have been reported at concentrations above 19.0 mM, due to greater toxicity of these oxyanions at higher concentrations [6]. Thus, the non-toxic doses <12.5 mM were chosen for understanding the mechanistic aspects of extracellular SeO_3_^2**−**^ reduction and biomimetic synthesis of elemental Se° nanospheres in this study using the spent medium (culture supernatant) of *P. aeruginosa* strain JS-11.

### Phenazine-1-carboxylic Acid Production and Reducing Activity in Culture Supernatant

**Figure 2:**
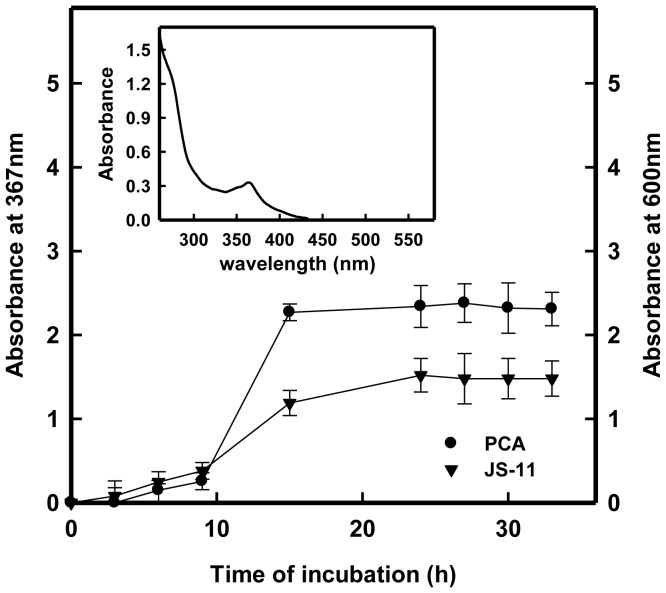
Growth curve and PCA production by strain JS-11. Bacterial growth in LB and the PCA present in culture supernatant were measured at 600 and 367 nm, respectively, as a function of time. Inset shows the absorption spectra of PCA with λmax at 367 nm, as determined by UV-visible spectrophotometer. The data represent the mean ± S.D of two independent experiments done in triplicate.

**Figure 3:**
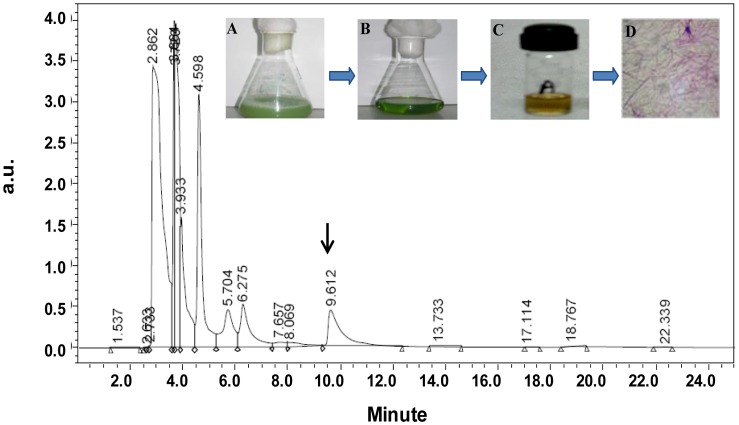
HPLC analysis of PCA produced by the strain JS-11. HPLC profile indicating the PCA peak at retention time of 9.6 min. Inset shows the (A): bacterial culture supernatant, (B): Benzene extract of PCA, (C): PCA after extraction and (D) PCA crystals.

**Figure 4:**
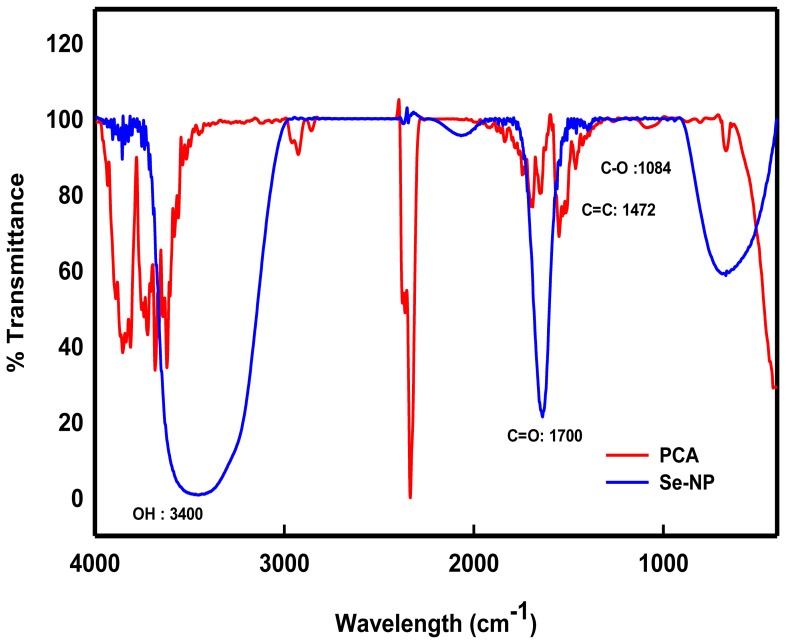
FTIR analysis of PCA. The spectra depict the changes in the peaks of PCA alone (red) and after treatment with 2 mM Na_2_SeO_3_^2**−**^ solution (blue).

Fig. 2 shows the growth curve of *P. aeruginosa* strain JS-11 and release of a redox active metabolite phenazine-1-carboxylic acid (PCA) in LB medium at 600 and 367 nm, respectively. Significant PCA synthesis occurred in the exponential phase, and reached to plateau at the stationary phase of bacterial growth (Fig. 2). PCA produced by strain JS-11 was crystallized in 0.1 N NaOH and the absorbance spectra of the yellow colored PCA solution in ultrapure water was obtained in the range of 250–550 nm (Fig. 2, inset). The characteristic absorption peak of PCA was obtained at 367 nm, which is identical to the PCA absorption wave length reported by Maddula et al. [40]. The PCA released in bacterial culture was further confirmed by HPLC analysis of PCA crystals dissolved in ultrapure water (Fig. 3). A typical peak appeared at a retention time of 9.6 min represents PCA, which conforms to earlier report [32], and reaffirmed the presence of this reducing agent in the culture supernatant. Incubation of 100 µl PCA solution with 2 mM Na_2_SeO_3_^2**−**^ resulted in appearance of red color Se° (Se-NPs) within 2 h. The data shown in Fig. 4 provide the information on the FTIR analysis of PCA alone and in presence of 2 mM Na_2_SeO_3_^2**−**^. The FTIR spectra exhibited two clear peaks at 1,700 and 3400 cm**^−^** ^1^, whereas the absorbance peaks of PCA appeared at 1,693.8 and 1,605.7 cm**^−^** ^1^ (Fig. 4). The shift in O–H broad absorbance peak between 3,000 and 3,500 cm**^−^** ^1^ in the FTIR spectra of PCA indicated significant modification of PCA molecules upon interaction with Na_2_SeO_3_^2**−**^ and its consequent reduction into Se-NPs. Moreover, the disappearance of PCA peaks at 1084 cm^–1^ suggests the occurrence of coordination between oxygen atom in the –C–O–C group of PCA and the Se atom.

**Figure 5:**
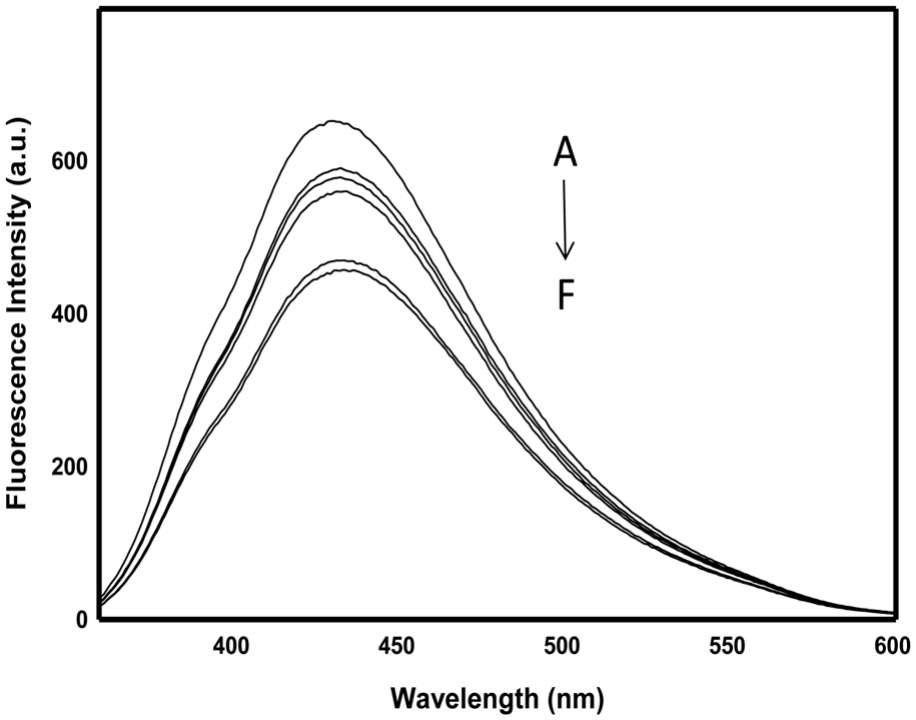
NADH fluorescence of bacterial supernatant alone and after treatment with NPs. The arrow represents the fluorescence quench of spectra A–F, where A is untreated control supernatant, and spectra B to F represent the supernatant treated with TiO_2_-NPs, ZnFe_2_O_4_-NPs, CdS-NPs, Ag-NPs (100 µgml**^−^** ^1^) and EMS (2 mM) in a total volume of 3 ml, respectively.

Also, the reducing potential of the cell-free culture supernatant, based on KMNO_4_ oxidation through the back titration assay was determined to be 1 mgml**^−^** ^1^ in LB medium (data not shown), which corresponds with the reduction ability of *K. pneumoniae* (0.96 mgml**^−^** ^1^) in LB medium [33]. Indeed, the reduction ability of cell-free culture supernatant varies with the types of culture media used for growing the organisms [33]. The reducing potential of the supernatant was further validated by measuring the native fluorescence of extracellular nicotinamide adenine dinucleotide (NADH) released by strain JS-11 in culture medium (Fig. 5). The fluorescence of NADH was observed to be quenched by 9.0, 10.8, 13.25, 27.0 and 29.1% with the addition of TiO_2_-NPs, ZnFe_2_O_4_-NPs, CdS-NPs, Ag-NPs and EMS, respectively. These results signify the interaction of metal NPs with NADH, and suggested that the extracellular NADH could also be one of the important factors in reducing SeO_3_^2**−**^ to elemental Se^0^. NADH is known to play a fundamental role in the conversion of chemical energy to useful metabolic energy [39], [41]. It is a well known reduced co-enzyme in redox reaction, and can be used as reducing agents by several enzymes [42], [43]. Thus it is concluded that the reducing activity of strain JS-11 culture supernatant is mainly attributed to soluble redox active agents like PCA and NADH, besides the already known NADH dependent reductases.

### Extracellular Biosynthesis of Se-NPs

**Figure 6:**
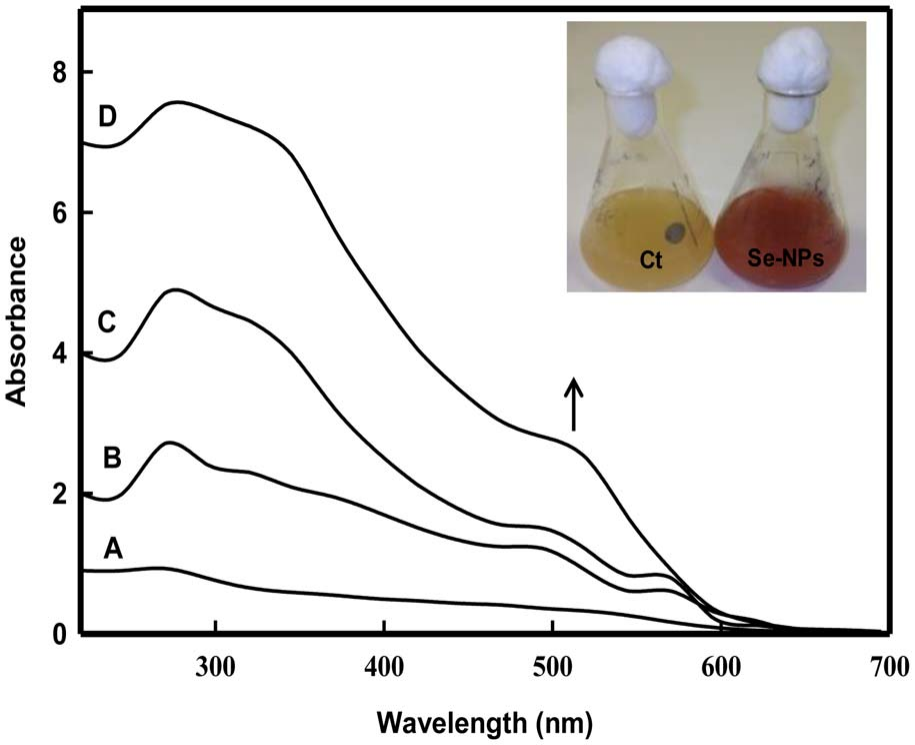
UV-Visible absorption spectra of extracellularly synthesized Se-NPs. The typical surface plasmon resonance (SPR) band is shown at 520 nm. The labels A–D represent 2, 24, 48 and 72 h of incubation, respectively. Inset depicts the change in color of culture supernatant from pale yellow to red after 24 h of incubation with 2 mM Na_2_SeO_3_^2**−**^ solution.

The culture supernatant of bacterial strain JS-11 when challenged with 2 mM Na_2_SeO_3_^2**−**^ solution, exhibited a change in color of the solution from light yellow to red (Fig. 6). The appearance of the red color indicated the occurrence of the reaction resulting in the formation of elemental Se° in solution. The characteristic red color of the reaction solution was due to excitation of the surface plasmon vibrations of the Se-NPs and provided a convenient spectroscopic signature of their formation [44]. The synthesis of Se-NPs in solution was monitored by measuring the time dependent changes in the onset absorbance at an interval of 2, 24, 48, and 72 h. The onset absorption of NPs was centered at 520 nm (Fig. 6) with a red shift with increasing time period. A time dependent increase was noticed in the absorption peaks of Se-NPs. No absorption peak corresponding to the control supernatant (without Na_2_SeO_3_^2**−**^) or Se ion solution in the range of measurement was observed. Also, the symmetric plasmon band implies that the solution does not contain much of aggregated particles.

The application of the biological systems for synthesis of Se-NPs has been reported earlier [21], [45]. However, the exact reaction mechanism leading to the formation of Se-NPs by the organisms has not yet been elucidated. Earlier studies suggested that NADH and NADH dependent nitrate reductase enzyme are important factors in the biosynthesis of metal nanoparticles. Ahmad et al. [46] reported that certain NADH dependent reductases are involved in reduction of metal ions in case of *F. oxysporum*. Also, Wang et al. [29] suggested the reduction of metal ions by the nitrate-dependent reductase and a shuttle quinine extracellular process. The reductases may also function as a capping agent, and ascertain the formation of thermodynamically stable nanostructures [47]. Hunter and Manter [21] have demonstrated the presence of selenite reductase (∼ Mol. Wt. 115 kD) and a 700 kD protein capable of reducing both selenate and nitrate in cell free extracts of *Pseudomonas* sp. strain CA5. Etezad et al. [20] have also reported the role of NADPH and NADPH-dependent selenate reductases in SeO_4_^2−/^SeO_3_^2**−**^ reduction. Therefore, it is likely that a multi-component redox system including PCA, NADH and most likely NADH-dependent reductases in the culture supernatant of strain JS-11 might act independently and/or in conjunction in catalyzing the biomimetic synthesis of Se-NPs in aqueous medium.

### Physical Attributes of Se-NPs

**Figure 7:**
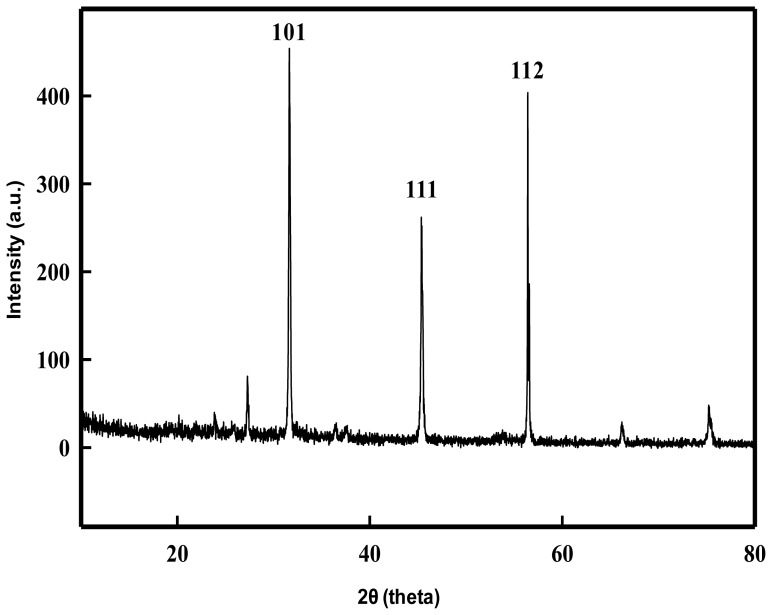
XRD pattern of the bacterial Se-NPs. The characteristic strong diffraction peak located at 31.64° is ascribed to the (101) facets of the face-centred cubic elemental Se° structure.

**Figure 8:**
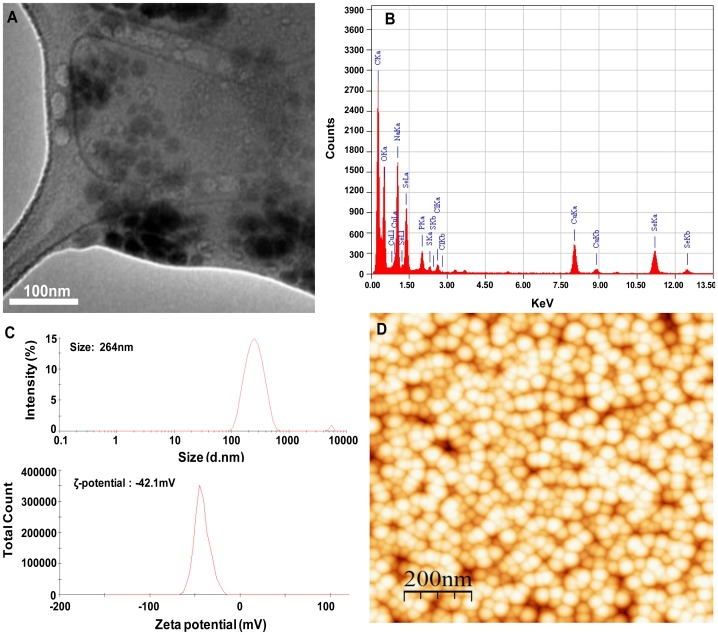
Microscopic analysis of Se-NPs produced by strain JS-11. Panel (A) shows the representative transmission electron micrograph recorded from a drop-coated film of the aqueous solution of Se-NPs; Panel (B) represents the energy dispersive X-ray spectrum of Se-NPs; (C) represents the average hydrodynamic size and zeta potential of Se-NPs; and (D) represents the 3D topography of Se-NPs in top view (scan size is 5×5 µm) by atomic force microscopic analysis.

The XRD pattern obtained for the extracellular Se-NPs with three intense peaks in the whole spectrum of 2θ values ranging from 20 to 80 is shown in Fig. 7. The diffractions at 31.64°, 45.35° and 56.41° can be indexed to the (101), (111) and (112) planes of the face-centered cubic (fcc) Se-NPs, respectively. The lattice parameters calculated by the Powder × software revealed that the maximum deviation that occurred between the observed and calculated values of interplanar spacing (d) remains below 0.002 Å. The full-width-at-half-maximum (FWHM) values measured for 101 planes of reflection were used to calculate the size of the NPs. The calculated average particle size of the extracellularly produced Se-NPs was determined to be 21 nm. A representative TEM image recorded from Se-NPs film deposited on a carbon-coated copper grid is shown in Fig. 8(A). The image shows the individual Se° particles as well as some aggregates. The morphology of the Se-NPs was predominantly spherical. The EDS spectra derived from a nanosphere indicated that it was composed entirely of selenium (Fig. 8B). The Cu peaks were associated with the TEM grid, the Na and Cl peaks reflected the high salt content of the medium, and the C and O peaks most likely were associated with cellular exudate. The lack of any other metal peaks in the spectrum suggested that the selenium occurred in the elemental state Se° rather than as a metal selenide (Se^2**−**^).The results of hydrodynamic size of the Se-NPs obtained with dynamic light scattering are shown in Fig. 8 (C). The distribution curves show the Se-NPs aggregates of 264 nm in deionized ultrapure water. DLS is widely used to determine the size of brownian NPs in colloidal suspensions in the nano and submicron ranges. The higher size of nanoparticles in aqueous suspension as compared to TEM size might be due to the tendency of particles to agglomerate in aqueous state. The results also corroborate with the finding of Tran and Webster [48]. Since, the primary and secondary sizes of the NPs are regarded as important parameters, therefore, the behavior of Se-NPs in MQ water was evaluated through dynamic light scattering (DLS), to understand the extent of aggregation and secondary size of these NPs. However, the zeta potential of Se-NPs in aqueous solution was estimated to be −42 mV (Fig. 8C). The large negative or positive zeta potential indicates the repulsion between particles with little tendency to come together. On the contrary, if the particles have low zeta potential values then there is propensity of particles to come together and form aggregates. Thus, the high negative charge on Se° nanospheres is probably responsible for their higher stability without forming very large aggregates over a prolonged period of time. The morphology and size of the nanoparticles were further validated by AFM analysis. Fig. 8(D) shows the AFM image of Se-NPs obtained on scanning probe microscope in tapping mode, under ambient conditions. The average size of the nanoparticles and roughness (Ra) of surface were determined to be 23 nm and 18 nm, respectively using the WSXM and SPIP softwares.

### Se-Assay for Nanotoxicity Assessment

**Figure 9:**
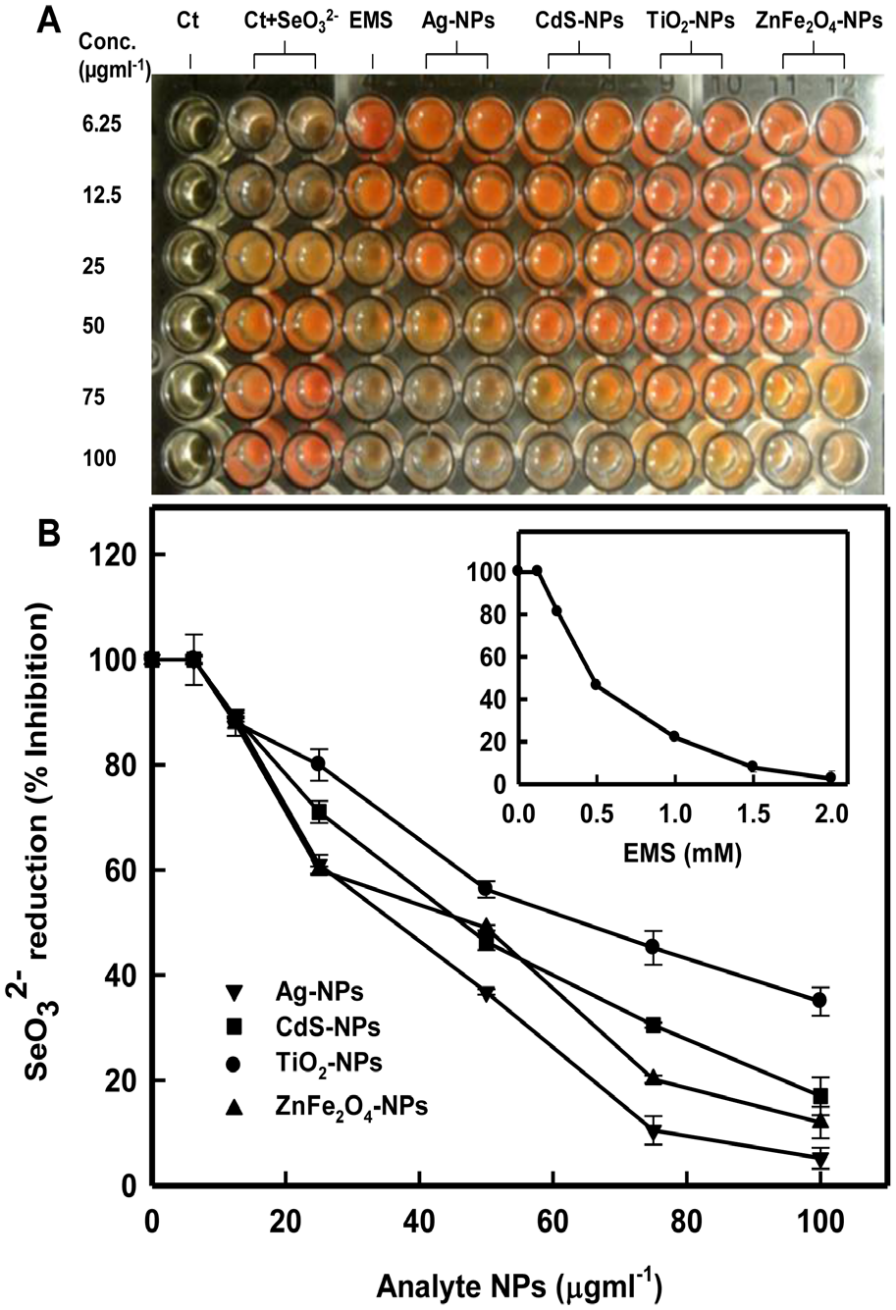
Se-Assay for toxicity assessment. Panel A: Colorimetric determination of toxicity based on inhibition of the reduction of SeO_3_^2**−**^ (colorless solution) to Se° (red) in absence and presence of toxic analyte NPs. Lane 1: Control (untreated supernatant); Lane 2 and 3: Control (supernatant +20 mM SeO_3_^2**−**^); Lane 4: positive control (EMS 0.125 to 2 mM); Lanes 5–12: Ag-NPs, CdS-NPs, TiO_2_-NPs and ZnFe_2_O_4_-NPs, in duplicate wells in 96 well microtitre plate. The density of the red color was read at 520 nm on multi-well microplate reader. Panel B: Percent inhibition of reduction in SeO_3_^2**−**^ to Se° in presence of analyte NPs. Inset shows the percent inhibition of SeO_3_^2**−**^ reduction by EMS, a known genotoxicant.

A simple bacterial cell-free bioassay (Se-assay) was developed based on the reduction of SeO_3_^2**−**^ to a red colored elemental Se° in culture supernatant of metabolically competent bacterial strain JS-11 cells. The Se-assay could be effectively used for visual assessment of the relative toxicity of a variety test chemicals including nanomaterials. The assay is based on the ability of a toxicant to inhibit the innate reducing power of the bacterial supernatant, and thereby impedes the SeO_3_^2**−**^ reduction to Se^0^. It is a phenomenon that does not occur in culture supernatant of dead or compromised cells, which maintains the original transparent and colorless state of SeO_3_^2**−**^ solution. The development of red colored elemental Se° upon SeO_3_^2**−**^ reduction is attributed either due to the PCA, NADH or NADH reductases, released in the culture supernatant, and regarded as metabolic markers. The data in Fig. 9 revealed the percent inhibition of SeO_3_^2**−**^ reduction at the highest concentration (100 µgml**^−^** ^1^) of the Ag-NPs, ZnFe_2_O_4_-NPs, CdS-NPs, and TiO_2_-NPs as 94.8%, 88%, 83% and 65%, respectively, which has suggested the differential toxicity of analyte NPs in the order as Ag>ZnFe_2_O_4_> CdS>TiO_2_. The IC_50_ values based on the concentration of NPs that inhibits 50% of the SeO_3_^2**−**^ reduction were determined to be 35, 47, 47 and 63 µgml**^−^** ^1^ for Ag-NPs, ZnFe_2_O_4_-NPs, CdS-NPs and TiO_2_-NPs, respectively. The percent inhibition with a known environmental toxicant EMS was observed to be 97.5% with an IC_50_ value of 24 µgml**^−^** ^1^. The IC_50_ or EC_50_ values vary with the nature of NPs and the assay types used for the assessment. Aruoja et al. [49] reported the EC_50_ values of ZnO-NPs, TiO_2_-NPs and CuO-NPs to *Pseudokirchneriella subcapitata* in algal growth inhibition test as 0.04 mgl**^−^** ^1^, 5.83 mgl**^−^** ^1^ and 0.71 mg l**^−^** ^1^, respectively. Whereas, the EC_50_ value of Ag-NPs has been reported to be 45–47 mgl**^−^** ^1^ in *Vibrio fischeri*, based on inhibition of bioluminescence [50]. Thus, any comparisons of IC_50_ or EC_50_ values will be difficult due to differences in sensitivities of the assay systems. Nevertheless, it is suggested that in Se-assay, the SeO_3_^2**−**^ reduction is closely linked to the metabolic status of a bacterial cell and regarded as a measure of cell viability and integrity, which could be qualitatively or quantitatively determined based on the intensity of the red colored Se° product. Since, more active cells are able to generate and release more reducing factors in culture supernatant, therefore, a more intense red color develops due to greater reduction of SeO_3_^2**−**^ to Se° [21]. Whereas, the treated cells or their culture supernatant under the influence of toxic effects of NPs or any other toxicant may lose their SeO_3_^2**−**^ reduction ability and remains colorless. Thus, the Se-Assay entails a sharp red or colorless output, which could be easily applicable for prescreening of a variety of environmental toxicants including nanoparticles prior to intensive toxicity investigations.

### Conclusions

In this study, the Se-NPs were synthesized employing the green technology, which involves the biological reduction process by the SeO_3_^2**−**^ tolerant bacteria *Pseudomonas aeruginosa* strain JS-11. The strain JS-11 maintains the characteristics encompassing the (i) rapid and easy growth with greater tolerance to higher concentrations of SeO_3_^2**−**^, (ii) capability of viable cells to perform the intracellular and extracellular reduction of SeO_3_^2**−**^ to elemental Se^0^, (iii) susceptibility to target analytes, such as metal NPs and EMS, and (iv) ability of non-viable or dead cells to lose the ability of SeO_3_^2**−**^ reduction. The Se-NPs were characterized by UV-Vis, XRD, TEM, EDX, FTIR and AFM analyses. It is envisaged that the metabolically active culture supernatant of SeO_3_^2**−**^ tolerant strain JS-11 can be exploited as a redox active system for economically viable and environmental friendly production of Se-NPs. Furthermore, it is elucidated that the formation of red Se° from SeO_3_^2**−**^ could serve as a molecular marker, whereas the inhibition of critical bioreduction step was considered as a toxicity end point for the qualitative and quantitative toxicity assessment. Nevertheless, further studies are warranted to better understand the stoichiometry of SeO_3_^2**−**^ reduction as function of temperature and pH, to optimize the efficiency of Se-NPs production. Also, the Se-assay needs further validation with known xenobiotics comprising a wider spectrum of toxicants, to firmly establish this method as a broad spectrum and low cost eco-toxicity assay for pre-screening of an array of environmental toxicants.

## Supporting Information

Figure S1 **Linear relationship between the red colored elemental Se**° **(Se-NPs) formed on reduction of SeO_3_^2−^, as a function of SeO_3_^2−^ concentration, using the software Sigma Pot 10.0.**
(TIF)
