# Supplementary figures and images for: Biomimetic Synthesis of Selenium Nanospheres by Bacterial Strain JS-11 and Its Role as a Biosensor for Nanotoxicity Assessment: A Novel Se-Bioassay
Source: PLoS One. 2013 Mar 4;8(3):e57404. doi: 10.1371/journal.pone.0057404 (PMC3587581; doi:10.1371/journal.pone.0057404)

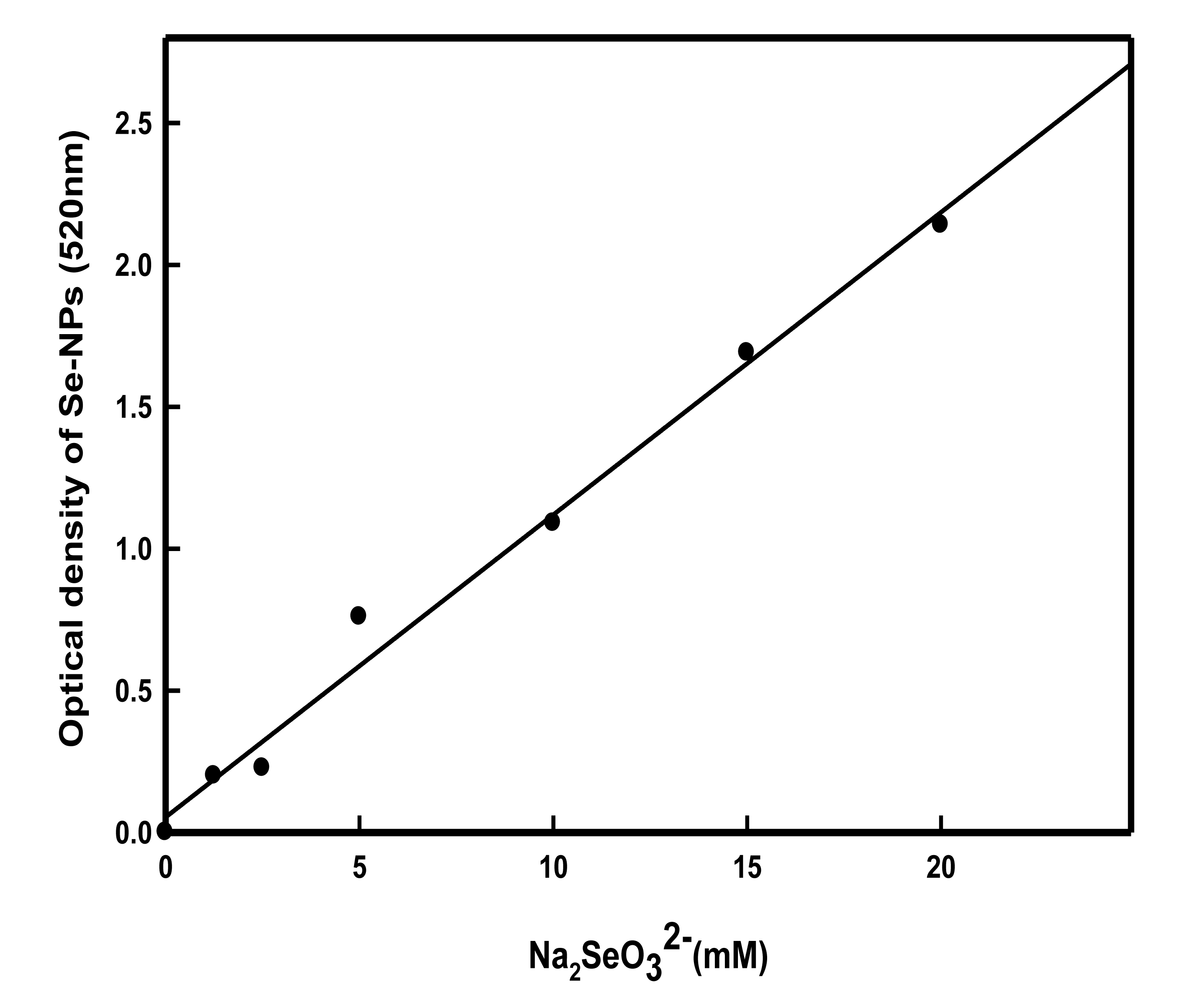

Supplement: Figure S1 — Linear relationship between the red colored elemental Se° (Se-NPs) formed on reduction of SeO32−, as a function of SeO32− concentration, using the software Sigma Pot 10.0. (TIF) [file pone.0057404.s001.tif]
